# Software-Defined NB-IoT Uplink Framework—The Design, Implementation and Use Cases

**DOI:** 10.3390/s21248234

**Published:** 2021-12-09

**Authors:** Alicja Olejniczak, Olga Błaszkiewicz, Krzysztof K. Cwalina, Piotr Rajchowski, Jarosław Sadowski

**Affiliations:** Faculty of Electronics, Telecommunications and Informatics, Gdańsk University of Technology, 80-233 Gdańsk, Poland; alicja.olejniczak@pg.edu.pl (A.O.); olga.blaszkiewicz@pg.edu.pl (O.B.); piorajch@eti.pg.edu.pl (P.R.); jaroslaw.sadowski@eti.pg.edu.pl (J.S.)

**Keywords:** framework, NB-IoT, academic testbed, simulations

## Abstract

In the radiocommunication area, we may observe a rapid growth of new technology, such as 5G. Moreover, all the newly introduced radio interfaces, e.g., narrowband Internet of Things (NB-IoT), are strongly dependent on the software. Hence, the radiocommunication software development and optimization, as well as the 3GPP technical specification, should be introduced at the academic level of education. In this paper, a software-defined NB-IoT uplink framework in the field of design is presented, as well as its realization and potential use cases. The framework may be used as an academic tool for developing, investigating, and optimizing the digital transmitter paths. The proposed realization is focused on the key elements in the physical layer of the NB-IoT interface used in the sensor devices. Furthermore, the paper also highlights the need of the data processing optimization to minimize the power consumption and usage of the resources of the NB-IoT node during transmitting gathered telemetric data.

## 1. Introduction

Sensors and sensor networks are nowadays identified as a ubiquitous solution present in everyday life. The development of the Internet of Things (IoT) is rapid, mainly due to the demand for automation and constant monitoring. The large amount of data generated by the IoT devices determines the need for network and data processing optimization and further designing a global solution for large business entities. In general, the scientific research papers involving the IoT networks may be divided into various academic branches of knowledge, such as radiocommunication, informatics, or electronics.

The new technology introduced in the 5G networks, where the narrowband Internet of Things (NB-IoT) interface is assumed to be present [1], is mostly based on the digital signal processing. The elements of the core network are designed to be the virtual components, and some parts of the terminals may be realized digitally. It seems natural to propose a software-defined framework that will provide the functionality of the NB-IoT terminal with the internal operations split into functional modules, which is the main goal of the conducted research. Decomposition of the uplink or downlink path gives an ability to distinguish educational and potential research and development possibilities. The proposed framework may also play the role of a novel laboratory testbed (even totally virtual), as a part of the software-defined radio (SDR) laboratory stand, including the radio frequency (RF) measurement equipment or over the air (OTA). The NB-IoT signals generated based on proposed framework may be verified regarding reference vectors, software base stations (BSs) simulators, or measurement devices.

In the presented framework, the signal processing path for the the NB-IoT uplink interface is implemented. The proposed modular structure of the software may support the learning process, including both advanced aspects of radiocommunication and programming in general. Additionally, such a framework is a fine tool to teach students how to extract theoretical assumptions based on the technical specification (e.g., the 3GPP technical documents) and further implement them. The advantage of the framework is its operation in the physical (PHY) layer of the NB-IoT interface, with low-level decomposition, which gives the ability of using simple and flexible tools for evaluation of the student implementations, without any auxiliary advanced protocol analyzers, interpreters, or even commercial licensed software.

The rest of the paper is structured as follows. The general overview of the NB-IoT interface is provided in Section 2. In Section 3, the related works and similar approaches are described. In Section 4, the proposed software-defined framework structure is presented. In Section 5, the uplink physical layer modular implementation in accordance with 3GPP standardization is proposed, while in Section 6, the analysis of the obtained results is presented. The paper is concluded briefly in Section 7.

## 2. The NB-IoT Interface

The NB-IoT standard [2] is directly connected with 4G technology, especially the physical layer. The number of signal processing operations of the uplink and downlink Orthogonal Frequency Division Multiple Access/Single Carrier Frequency Division Multiple Access (OFDMA/SC-FDMA) techniques is definitely challenging, bearing in mind the signal formation computational cost and the low complexity of the nodes. The NB-IoT device lifespan should reach at least 10 years [3], and because of that, the software design, especially considering repetitive operations, should be computationally effective. Unfortunately, the obligatory compliance with the 3GPP standard dismisses the ability to omit the most complicated operations (by simplifying the radio interface) as per in the typical industrial, scientific, and medical (ISM) sensor networks. The NB-IoT is a cellular network standard developed by the 3rd Generation Partnership Project (3GPP) organization in 2016 [4]. The physical layer mostly inherits from the long-term evolution (LTE), e.g., the OFDMA technique for downlink, the SC-FDMA technique for uplink, the channel coding, the interleaving operation, and functioning in the licensed spectrum [2,3]. All the assumptions regarding the NB-IoT interface were published for the first time in Release 13 of the 3GPP standard. The fact that the NB-IoT interface is based on LTE allows reducing the final cost of the device as well as the system deployment time [5]. The main goal of the NB-IoT is to provide the communication between devices in harsh radio conditions, e.g., underground or in basement environments [6,7,8].

Coverage enhancement defined in the NB-IoT specification gives 20 dB higher maximal coupling loss with respect to the Global System for Mobile Communication (GSM) packet data service [9,10]. Nevertheless, apart from abovementioned features, there are other advantages of the NB-IoT usage, e.g., energy efficiency or an easy integration with the preexisting cellular networks. NB-IoT provides two types of energy saving modes, i.e., power-saving mode (PSM) and extended/enhanced discontinuous reception mode (eDRX) [11], that may increase user equipment (UE) battery lifetime up to 10 years. Considering coexistence with other cellular systems, it is worth mentioning that the NB-IoT system may operate in three different modes, determining the NB-IoT physical resource blocks (PRBs) deployment within the current spectrum, i.e., standalone, in-band, and guard-band [12]. According to the in-band and guard-band modes, the NB-IoT PRBs (each occupying 180 kHz of spectrum—12 subcarriers with 15 kHz spacing or 48 subcarriers with 3.75 kHz spacing) are allocated inside the LTE resource grid. To be more precise, the NB-IoT signal may occupy the unused PRB of LTE bandwidth (in-band) or PRB of LTE guard bands (guard-band). Alternatively, regarding standalone mode, NB-IoT PRBs may utilize the GSM channel as well [13]. Considering its bandwidth of 200 kHz, it is possible to allocate 180 kHz NB-IoT PRB with two additional guard buffers, 10 kHz each. The key differences of the NB-IoT interface with respect to LTE may be identified in the protocol stack, like the Hybrid Automatic Repeat Request (HARQ) and terminal modes of operation. By definition, the NB-IoT devices are dedicated for controlling or monitoring applications. Therefore, due to the assumed lack of nodes mobility, it not only reduces the amount of radio resources utilized in control channels but also eliminates the problem of handover between the evolved NodeBs (eNBs). It is worth mentioning that the maximal throughput reaches 200 kbps or 160–250 kbps [14,15] for uplink and downlink, respectively, and it is allocated only for the data transfer services.

## 3. Related Work

The SDR-based concept of modern radiocommunication systems is widely presented in the literature. Available powerful simulation environments, such as Matlab [16], Simulink [17], or Labview [18], provide toolkits for developing sample testbeds or analyzing the real radio signals. Each of the mentioned tools and methods may be used for didactic purposes, especially if the goal is to analyze the LTE protocol stack and the physical layer. Nevertheless, conducting the didactic or research activities, when the signals generated in the simulation environment must be processed in real time, e.g., for the SDR platform, is problematic. Furthermore, except for scientific and educational benefits that may be derived from simulation software usage, such tools may be questionable in the context of industry, where every part of software must meet the requirements of real hardware platforms and cannot contain licensed code from the third parties with licensing restrictions.

At the academic stage of education, the low-level programming and implementation should be definitely used to demonstrate the principles of basic radiocommunication operations and signal processing methods. In the state-of-the-art research studies, many examples of the IoT and NB-IoT networks may be found. Nevertheless, none of the presented examples allow decomposition of the basic operations within NB-IoT uplink and downlink enabling thorough study concerning low-level programming of signal processing procedures. According to the publications review, a few trends may be observed, where the main goal was to make the development of the NB-IoT networks simple and well investigated.

In [19], authors presented the scope of IoT simulators, e.g., the OMNeT++ and IOTSim, underlining the increase in the popularity of the IoT networks and the need for testing the proof-of-concept during the research studies. Although the paper [19] presents an extensive study of the simulators, they cannot be used for the NB-IoT interface decomposition due to the incompatibility with the standards. In [3,14], authors investigated the operation of the entire NB-IoT networks deployed on a wide area. Naturally, these frameworks are advanced in the scope of protocol layers analysis or even considering the energy efficiency of the NB-IoT nodes [3]; nonetheless, they are directed at scientific research rather than educational purposes, and cover neither the issue of the physical layer analysis nor NB-IoT software development.

In other research [20], authors presented a realization of a protocol simulator for the NB-IoT communication. The content of the paper is focused on the uplink physical layer, channel, and transmission scheduling. In the paper, authors presented the definition and organization of the Narrowband Physical Uplink Shared (NPUSCH) channel, as well as the transmission operation chain. Mentioned operations are compliant with [2,21] 3GPP standards and correspond to the framework described in the given paper, yet the final realization is different. The developed software [20], based on Matlab and Python languages, should be treated as a high-level programmed black box or the model for partial NB-IoT simulations, e.g., without the extensive studies of the SC-FDMA scheme or execution on several platforms.

In [16,22], the authors proposed a software-defined tool suitable for generating the NB-IoT downlink signal or complete uplink frames. Software described in [22] is based on the open-source LTE-Sim simulator [23]. It provides extensive LTE network simulations, including the physical layer functionalities, protocols development, and architecture optimization. Despite following Release 9 of the 3GPP standard, authors introduced additional functionalities dedicated to the NB-IoT interface that were included in Release 13. Nevertheless, the proposed tool was designed to simulate the entire network operation rather than analyze the particular communication layers. The paper [16] contains a description of a tool for generating the basic downlink synchronization signals only, excluding broadcast channel. Hence, this concept may be treated as the NB-IoT vector signal generator instead of integrated framework.

In [1], authors described the process and issues involving development of the NB-IoT network simulator. The paper identified the key differences between the NB-IoT and LTE as well as the presence of NB-IoT in the 5G network. Moreover, authors also indicated the need for differentiating the analytical model and the network evaluation solutions. The evaluation solutions, understood as the network simulators, must be based on two parts: the main simulator and the testbed or emulator. In [1], the network simulator was developed, based on the ns-3 LENA and following a modular programming paradigm. This approach allowed authors to adapt existing software to realize new simulation features. Nevertheless, the presented research refers to an ongoing project and, thus, does not cover all the NB-IoT-related procedures. It is important to mention that, apart from a few similarities between [1] and the approach presented in this paper, the proposed framework supports low-level programming abilities based on extracted and block-structured physical layer procedures with an auxiliary set of reference vectors to provide the proper testing tools for every stage of NB-IoT implementation.

Apart from the studies analyzing NB-IoT particularly, the didactic trends regarding programming education in general should be examined as well. The rapid expansion of the software itself into various branches of science determines the necessity of introducing the programming context within the academic courses. Furthermore, many researchers emphasize that this issue should concern the whole way of thinking rather than focus on specific syntax teaching [24,25]. Multiple papers indicate the importance of block-structured programming teaching, involving real projects and active learning techniques to effectively encourage students to acquire knowledge and to provide them with the wider perspective of the given topic [24,25,26,27]. The commercial market demands determine the additional aspects of the academic programming education, especially in the context of digital signal processing. Hence, more complex implementation issues should be also taken into account, e.g., testing or optimization and parallel computing [28,29,30,31].

The literature review shows that the decomposition of the NB-IoT framework into separate and independent modules is desired, from both scientific and educational points of view. Since each functional module may be investigated separately, students may focus more precisely on the logical operations, which may improve the whole didactic process [32]. Such methodology is widely spread due to the growing popularity of information and communications technology (ICT) and technology in general. The mentioned didactic approach gives the possibility of adjusting the academic process with regard to students’ capabilities and dynamic changes in technology [33,34]. It allows students to implement their own functional modules, also as the Hardware Description Language (HDL) Intellectual Property (IP) cores for the Field-Programmable Gate Arra (FPGA) chips, and further test and optimize them. In the literature, the power consumption minimization directly related to optimal software implementation is stressed as well. Unfortunately, in the publications such as [35], authors were focused on investigating the modes of operations described in the standard, not the NB-IoT software implementation itself, due to the usage of the commercially available NB-IoT modems. As a consequence, no extensive modifications of the modem operation were allowed and possible. The provided framework eliminates this limitation and ensures a fully adjustable implementation of the transmitter path. Furthermore, incorporating NB-IoT systems, currently and widely used in the real world, may be an incentive for students to learn more willingly, as the main project goal is practical and explicitly defined [27]. Finally, placing a project on the 3GPP standardization grounds allows introducing work with technical documentation to the academic environment, and the reference open-access dataset containing the radio signals recorded during other experiments [36] may gain usability.

## 4. Framework Design

The proposed software-defined NB-IoT framework should be understood as an evaluational platform, where the signal processing functional blocks of the NB-IoT Rel. 13 and Rel. 14 standards are represented as a set of modules. It means that the design of the radio interface is completely modular, thus the adaptive form makes it possible to implement, execute, and test each part independently of the target platforms, testbeds, OTA stands, simulation environments, hardware–software stands with the FPGA support, or even distributed laboratory environments.

To achieve such versatility, software implementation of all modules must be independent, bearing in mind the specificity of the processor architecture and the method of random-access memory (RAM) addressing on the target platform. Thanks to this, it is possible to run these modules on the so-called embedded systems based on a microprocessor with the Advanced RISC Machine (ARM) architecture, as well as on computers with the x86-64 processors. Thus, it can be realized on almost any available hardware, even on the low-computational-power ones—dependent on the stands available in the laboratory. Therefore, framework is developed mostly in the procedural C (with elements of the C++, e.g., for the logging handling) language which also allows achieving the compatibility with the standard by means of the timing constraints. This element is crucial in the educational process—however, very often neglected during the studies and learning progress, where low-level programming knowledge is a key in designing, developing, and optimizing the radiocommunication systems and networks. This knowledge is extremely desirable by the industry from the specialists.

It is known that in order to implement an efficient and effective radiocommunication system, it is crucial to distinguish and potentially optimize (e.g., by parallelization approach) the calculations of the individual program modules. The mathematical calculations that will be performed for the longest time, compared to the other NB-IoT protocol stack modules, may be one of the examples. Such elements potentially include, e.g., channel encoding and decoding, time and frequency synchronization, channel estimation, or the Orthogonal Frequency-Division Multiplexing (OFDM) signal formulation. Evaluation of the efficiency of the signal processing in the individual section of the radio interface through measurements of the time stamps and determination of the execution time of the individual elements of the transceiver is an important parameter during developing of the radio interface—especially when it will be used in the sensor device.

The mentioned parallel processing of the radio signals requires the implementation of the multithreaded environment determining, e.g., the scope of parallelization in the radio interface structure. Thus, description and implementation of the modules should include not only the technical requirements resulting from the 3GPP standard, but also the possibilities of process optimization manner. As an example, the proper decomposition of the NB-IoT stack, which is proposed for the uplink in Section 5, can be given. This creates a wide set of potential use cases of the proposed framework, in almost all academic stages and several study courses. In addition, the proposed framework design can be transformed from the centralized software manner to the hybrid architecture, where modules (separately or as a bundle) are developed as the hardware intellectual property (IP) cores or the hardware coprocessors. Even a distributed architecture can be proposed, where modules will be implemented independently, e.g., as the separate laboratory stands, or even remotely, where the generated samples will be sent to the SDR platform via the laboratory network. The remote availability and distributed architecture may especially be very useful during the remote teaching [37].

The radioinformatics term should be used in that case. Such an approach also requires the specific educational methodology in which the proposed framework architecture fits completely. It enables the development, optimization (single-thread and multithreaded software), and verification (by using provided test vectors or compatible radio receiver) of the particular PHY layer radio signal processing blocks. In addition, it can be also used as a tool for the electronic courses where the FPGA evaluation boards may be efficiently used for NB-IoT interface purposes.

**Figure 1:**
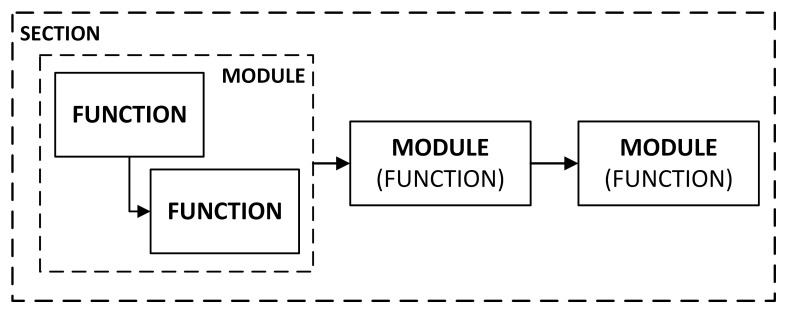
Proposed structures of the software components and their dependencies.

Therefore, the concept of separating the entire section in the NB-IoT radio interface architecture (the set of connected modules), individual modules, or individual functions inside these modules needs to be provided to improve the efficiency of the learning process and adjust the flexibility of the framework for the different courses. In Figure 1, the proposed structures of the software components and the relationships between them are shown.

According to the proposed structure of the framework, the module can be both the set of functions which process the datasets and a single function that will be further identified as the functional module. Each module obligatorily includes the input and the output vectors, the set of parameters, and references to the 3GPP or other publications with detailed signal processing description compliant with the NB-IoT stack protocol.

## 5. Uplink Implementation

**Figure 2:**
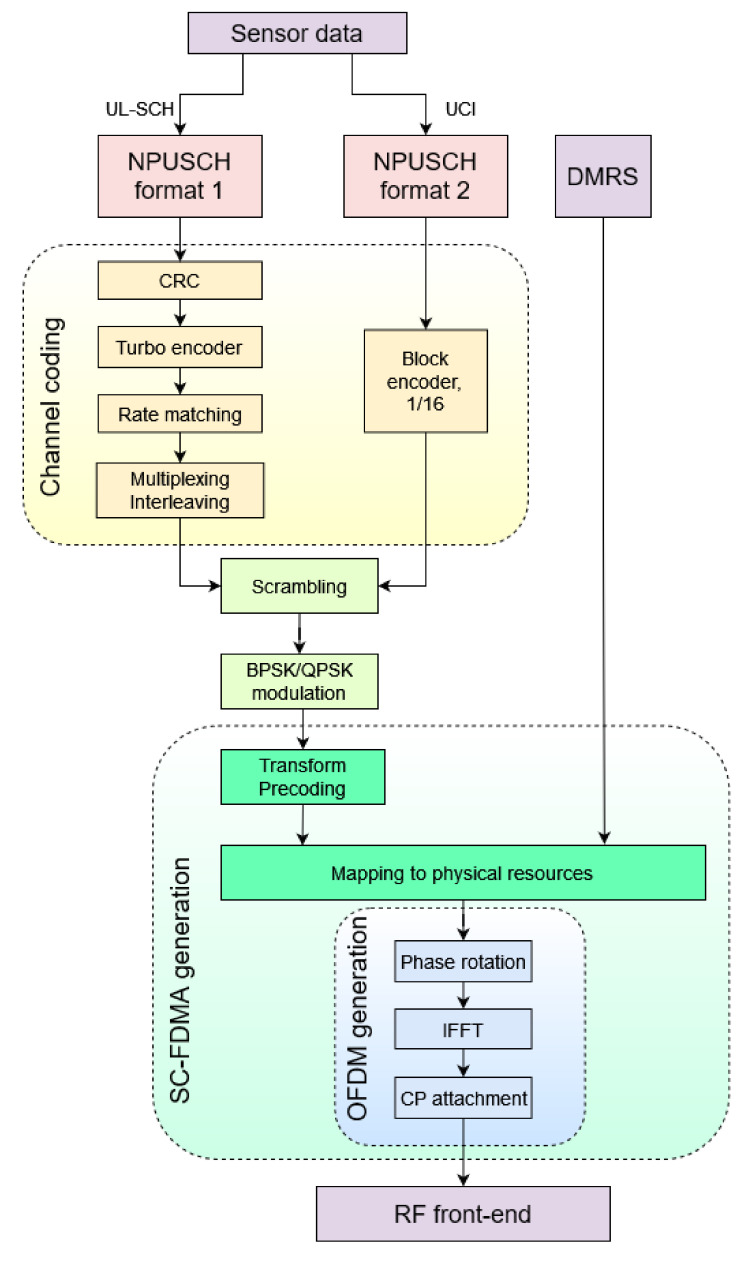
NB-IoT uplink modular procedures [2,21].

The following section presents consecutive modules that cover all the vital operations within the whole NB-IoT uplink framework, including both NPUSCH channel procedures and demodulation reference signal (DMRS) signals generation, in accordance with the 3GPP standards. Since the main concept is chunk-based, it is assumed that all transmitter blocks should be implemented and analyzed separately. However, holistic transmitter evaluation is inevitable as well. The modular architecture of the NB-IoT uplink procedures that fulfills the previously proposed approach is presented in Figure 2.

It is important to note that an uplink transmission may be processed based on the two different variants, i.e., format 1 or format 2, corresponding to the data and control information transmission, respectively. Each format determines applied channel coding procedures, whereas the further modules are uniform for both variants. All blocks presented in Figure 2, including the NPUSCH and DMRS paths, are described within the next subsections.

### 5.1. Channel Coding

The first section of the given framework provides the channel coding procedures. As the two possible NPUSCH formats are encoded differently, each processing module is described in the separate subsection. It is worth mentioning that the concept proposed by the authors allows treating the channel coding part twofold—as a single integrated instance or as a decomposed set of functions, depending on the educational goal.

#### 5.1.1. Cyclic Redundancy Check

Considering the fact that the DMRS symbols generation based on the initial parameters of the given transmission and one uplink control information (UCI) data packet in the NPUSCH format 2 contains one bit of control data only, the NPUSCH format 1 is crucial in the context of the sensor data acquisition and transmission. The forwarded data is divided and allocated to the transport blocks, for which transport block size (TBS) size is predetermined [38]. The given transport blocks are further conveyed consecutively to the first processing module of the channel coding section, i.e., cyclic redundancy check (CRC), providing error detection at the receiver side.

The CRC block extends an input vector for the 24 additional parity bits. The CRC polynomial is the following [21]:

(1) $$g_{CRC24A}=\left[D^{24}+D^{23}+D^{18}+D^{17}+D^{14}+D^{11}+D^{10}+D^{7}+D^{6}+D^{5}+D^{4}+D^{3}+D+1\right].$$

#### 5.1.2. Turbo Encoder

**Figure 3:**
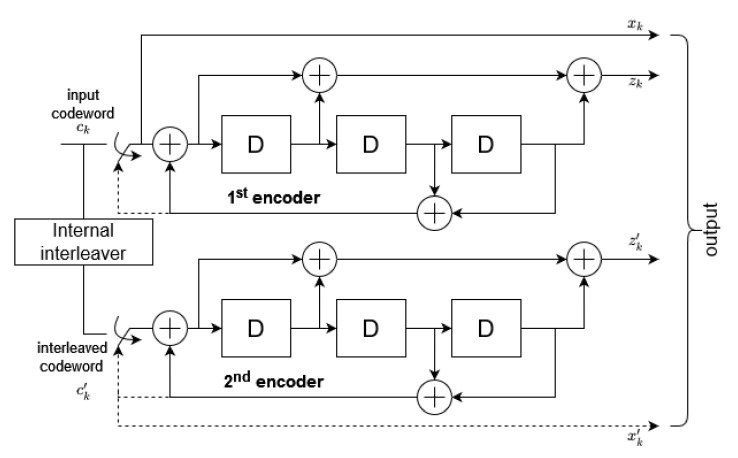
Turbo encoder with a rate of 1/3.

Thereafter, the output of the CRC module is transferred to the turboencoder with the 1/3 coding rate that is presented in Figure 3:

It is a combination of the two parallel shift registers (with *D* indicating consecutive delay blocks) that may be derived from the following transfer function:

(2) $$G\left(D\right)=\left[1,\frac{g_{1}\left(D\right)}{g_{0}\left(D\right)}\right],$$

where $g_{0}\left(D\right)=1+D^{2}+D^{3}$ and $g_{1}\left(D\right)=1+D+D^{3}$.

The original input data stream is shifted through the first register, whilst interleaved input bits are passed along the second one. The interleaving procedure is specified in [21]. The given encoder produces three bits for each bit of the transmitted codeword through $x_{k}$, $z_{k}$, $z_{k}^{\prime }$ streams and an additional 12 bits due to register reset, including also $x_{k}^{\prime }$ stream.

#### 5.1.3. Rate Matching

**Figure 4:**
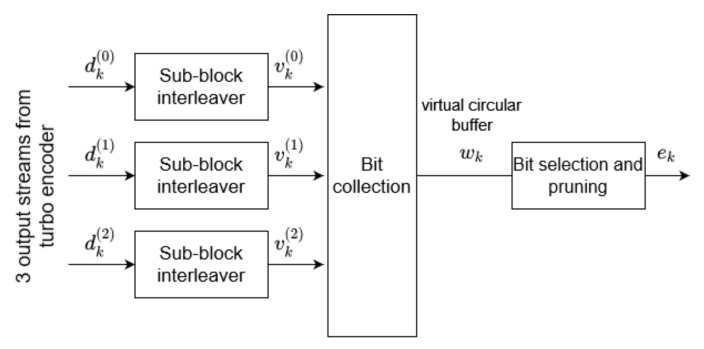
Rate-matching procedures.

The three output bit streams of the turbo encoder are further forwarded to the rate-matching block. The applied procedures are presented in Figure 4:

Initially, the parallel data streams are interleaved according to [21], resulting in product $v_{k}^{\left(0\right)}$, $v_{k}^{\left(1\right)}$, $v_{k}^{\left(2\right)}$ vectors, where $k=0,...K_{\Pi }-1$ and $K_{\Pi }=\left(R_{subblock}^{TC}\times C_{subblock}^{TC}\right)$. The number of columns $C_{subblock}^{TC}=32$ and number of rows $R_{subblock}^{TC}$ may be defined as the minimum integer fulfilling the formula

(3) $$D_{in}\leq \left(R_{subblock}^{TC}\times C_{subblock}^{TC}\right),$$

where $D_{in}$ represents the number of sub-block interleaver’s input bits. Subsequently, the $v_{k}$ streams are distributed within an $\left(R_{subblock}^{TC}\times C_{subblock}^{TC}\right)$ matrix, and an intercolumn permutation is applied. The permutation pattern is presented in Table 1.

Finally, the virtual circular buffer of length $K_{w}=3K_{\Pi }$ is formulated as follows:

(4) $$w_{k}=v_{k}^{\left(0\right)}$$

(5) $$w_{K_{\Pi }+2k}=v_{k}^{\left(1\right)}$$

(6) $$w_{K_{\Pi }+2k+1}=v_{k}^{\left(2\right)}.$$

The length of the output vector $e_{k}$ equals $E=Q_{m}\cdot G^{\prime }$, where $Q_{m}$ denotes the modulation order and $G^{\prime }=G/Q_{m}$ depends on the total number of bits available for the transmission *G*. The procedure of arranging bits within output vector $e_{k}$ is specified in detail in [21].

#### 5.1.4. Multiplexing and Interleaving

The last module of the channel coding section includes multiplexing and interleaving procedures. It should be noted that information data UCI is not processed within the given block. Hence, a multiplexing algorithm (Algorithm 1) may be reduced to the following form:
**Algorithm 1:** Reduced multiplexing algorithm.i,k=0 while i<G$g_{k}={\left[e_{i}\ldots e_{Q_{m}-1}\right]}^{T}$$i=i+Q_{m}$k=k+1 end while

Furthermore, the interleaver is applied in accordance with the 3GPP standard [21]. It is worth mentioning that at this stage of processing, the initial parameters of the NPUSCH transmission, e.g., the number of slots $N_{slots}^{UL}$, are taken into account. Hence, there are plenty of parameters and transmission variants to be tested, which determines the great number of the possible input/output vector sets to be verified. This may be a good opportunity to present the practical use of the unit testing to the students.

#### 5.1.5. NPUSCH Format 2—Block Encoder

The channel coding section for the NPUSCH format 2 includes the bit mapping only. Since the UCI data is transmitted via 1 bit, after the block coding module, it is represented by 16 identical bits. The procedure is presented in Table 2.

### 5.2. NPUSCH Parameters

At this stage of the uplink implementation description, it is important to introduce the basic transmission parameters that are predetermined by the higher stack layers before starting the NPUSCH communication. However, they should be already considered within channel coding calculations (e.g., estimation of the total number of the bits available for the transmission); from now, the context of the frame-based transmission is included in the performance of all modules. More explicitly, the channel coding section belonging to the uplink shared channel (UL-SCH) operates on the transport block concept, whereas the further NPUSCH procedures involve additional time-related factors with reference to the physical resources background.

As mentioned in Section 1, there are two possible variants of the NB-IoT subcarrier spacing: 15 kHz and 3.75 kHz. The number of available subcarriers and the time dependencies regarding those two parameters are presented in Table 3.

**Figure 5:**
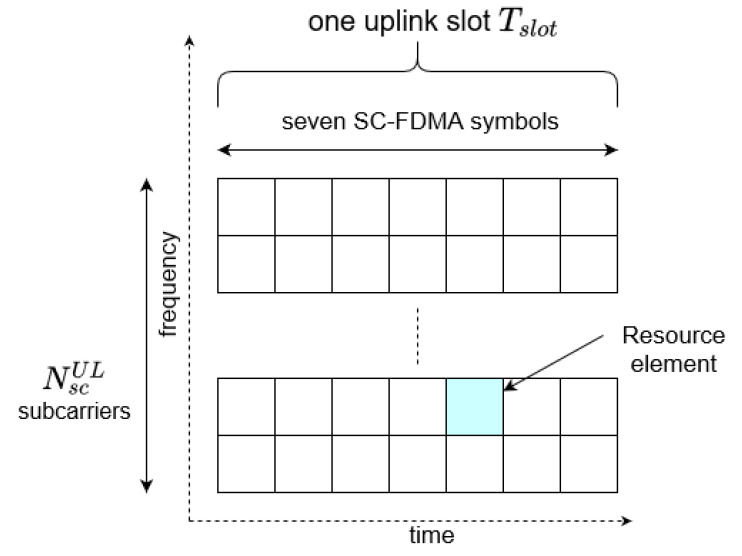
NB-IoT resource grid [2].

It is important to mention that $T_{slot}$ represents the time duration of a single slot, containing seven symbols (#0–6). To properly understand the slot term in the context of the transmitted bits, or more precisely, symbols and subcarriers, the exemplary NB-IoT resource grid is presented in Figure 5.

The 3GPP technical specification [2] defines possible combinations of the number of active subcarriers and slots as well, which are summarized in Table 4.

It should be noted that multitone mode is available only for the NPUSCH format 1 with 15 kHz subcarrier spacing, and $N_{sc}^{RU}$ indicates number of the active subcarriers for the resource uni (RU), i.e., all slots in the given transmission.

### 5.3. Scrambling

The first module of the NPUSCH, following the channel coding section, is scrambling. Apart from the fact that it is a separate module, it is also used within the other sections, e.g., during the DMRS pilots generation. In general, scrambling is based on the pseudorandom Gold sequence to provide the randomness within transmitted information [4]. The scrambling sequence generator shall be initialized with the following $c_{init}$:

(7) $$c_{init}=n_{RNTI}\cdot 2^{14}+n_{f}\;\mathrm{mod}\;2\cdot 2^{13}+\left\lfloor n_{s}/2\right\rfloor \cdot 2^{9}+N_{ID}^{Ncell},$$

where $n_{RNTI}$ denotes predetermined radio network temporary identifier, $N_{ID}^{Ncell}$ represents narrowband physical layer cell identity, $n_{f}$ constitutes system frame number, and $n_{s}$ indicates slot number within a radio frame. Furthermore, $c_{init}$ should be reinitialized after every $M_{identical}^{NPUSCH}$ that may be defined as follows:

(8) $$M_{identical}^{NPUSCH}=\left\{\begin{array}{cc} \min\left(\lceil M_{rep}^{NPUSCH}/2\rceil ,4\right) & N_{sc}^{RU}>1\\ 1 & N_{sc}^{RU}=1, \end{array}\right.$$

where $M_{rep}^{NPUSCH}$ denotes scheduled number of repetitions of the NPUSCH transmission [2]. The output vector of the scrambling module is the product of the XOR operation between the input bits and the calculated pseudorandom sequence. It is important to mention that the scrambling module operation is time-dependent in the context of continuous transmission, as the initialization factor $c_{init}$ is based on, e.g., the frame number that varies over time.

### 5.4. Modulation

The next module fulfills the function of mapping transmitted bits to the binary phase shift keying (BPSK) or the quadrature phase shift keying (QPSK) symbols. The mapping patterns for each modulation type are presented in Table 5 and Table 6.

The modulation pattern depends on the current parameters of the NPUSCH and is defined by the higher layers. In Table 7, some general limitations regarding modulation scheme are presented.

### 5.5. Transform Precoding

In order to mitigate the problem of the high peak-to-average power ratio (PAPR), the classical OFDM has been replaced by the SC-FDMA technique within the NB-IoT uplink transmission. The main difference between those two methods is the additional discrete Fourier transform (DFT) block that is implemented as a part of the transform precoding module. The detailed transformation scheme is described in [2], and it should be noted that the whole procedure encourages students to consideration combining both frequency and time domains.

### 5.6. Demodulation Reference Signals

**Figure 6:**
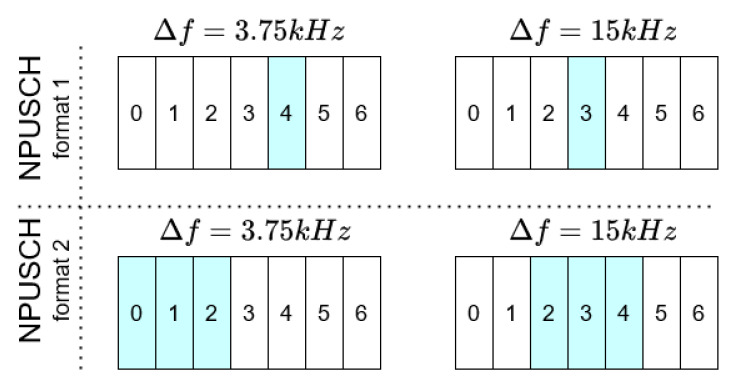
DMRS allocation [2].

The given module is responsible for the DMRS signal generation. Generally speaking, DMRS symbols are multiplexed with the NPUSCH symbols during the resource grid arrangement. The exact DMRS symbols allocation and the number of its occurrence depends on the parameters of the given transmission, as depicted in Figure 6.

The DMRS may be obtained based on the reference sequence $r_{u}\left(n\right)$ that varies in relation to $N_{sc}^{RU}$, as follows:

(9) $${\bar{r}}_{u}\left(n\right)=\frac{1}{\sqrt{2}}\left(1+j\right)\left(1-2c\left(n\right)\right)w\left(n\;\mathrm{mod}\;16\right),\;0\leq n<M_{rep}^{NPUSCH}N_{slots}^{UL}N_{RU}$$

NPUSCH format 1: $\;r_{u}\left(n\right)={\bar{r}}_{u}\left(n\right)$

NPUSCH format 2: $\;r_{u}\left(3n+m\right)=\bar{w}\left(m\right){\bar{r}}_{u}\left(n\right),\;m=0,1,2$

for $N_{sc}^{RU}=1$ and:

(10) $$r_{u}\left(n\right)=e^{j\alpha n}e^{j\phi (n)\pi /4},\;0\leq n<N_{sc}^{RU}$$

for $N_{sc}^{RU}>1$.

It should be noted that c(n) represents the pseudorandom Gold sequence described in Section 5.3, reinitialized based on $c_{init}=35$. The w(n), α and ϕ parameters are specified and tabularized in [2].

In order to divide the pilot sequences between the separate slots, the additional group-hopping parameter is introduced. Enabled group hopping provides the DMRS variety, due to changing *u* index that indicates the current value of both w(n) and ϕ for each NPUSCH slot.

In general, such a module with complicated calculations involving complex numbers that is used in real systems and thus is restricted by, e.g., the real-time operation limitations, may be a good didactic example demonstrating the importance of the computational effectiveness and reinforcing student awareness regarding connection between the implementation itself and its further practical use. Finally, it may be an effective exercise to develop good programming habits.

### 5.7. Mapping to Physical Resources

In general, the process of mapping transmitted data to the physical resources is based on arranging symbols within the resource grid presented in Figure 5. The given procedure should involve the current number of active subcarriers and map the symbols slot by slot, including the number of NPUSCH repetitions $M_{rep}^{NPUSCH}$. It is important to mention that within the slot for seven symbols, both NPUSCH and DMRS data should be arranged.

### 5.8. OFDM Generation

The last part of the NB-IoT uplink is the OFDM generation module. It should be noted that, taking into account the former transform precoding module (Section 5.5), the SC-FDMA signal is generated instead of the typical OFDM. Nevertheless, the previously presented modular approach of analyzing each procedure separately justifies such notation. The next subsections refer to the three main OFDM generation functions, i.e., the phase rotation, the inverse fast Fourier transform (IFFT), and the cyclic prefix (CP) attachment.

#### 5.8.1. Phase Rotation

In the case of $N_{sc}^{RU}=1$, the additional phase rotation $\phi_{k,l}$ of the transmitted symbols is applied, fulfilling the following requirements:

(11) $$\phi_{k,l}=\rho \left(\tilde{l}\;\mathrm{mod}\;2\right)+\varphi_{k}\left(\tilde{l}\right)$$

(12) $$\rho =\left\{\begin{array}{cc} \frac{\pi }{2}\; & for\;BPSK\\ \frac{\pi }{4}\; & for\;QPSK \end{array}\right.$$

(13) $$\varphi_{k}\left(\tilde{l}\right)=\left\{\begin{array}{cc} 0 & \tilde{l}=0\\ \varphi_{k}\left(\tilde{l}-1\right)+2\pi \Delta f\left(k+1/2\right)\left(N+N_{CP,l}\right)T_{s} & \tilde{l}>0 \end{array}\right.$$

(14) $$\tilde{l}=0,1,\ldots ,M_{rep}^{NPUSCH}N_{RU}N_{slots}^{UL}N_{symb}^{UL}-1$$

(15) $$l=\tilde{l}\;\mathrm{mod}\;N_{symb}^{UL}$$

where $T_{s}$ denotes the basic time unit 1/30,720,000 and *k* and *l* represent the subcarrier index and the SC-FDMA symbol consecutively.

#### 5.8.2. The IFFT

In the IFFT module, the inverse fast Fourier transform is performed simply over the obtained resource grid, involving the context of the slots and subcarriers as well. Depending on the current subcarrier spacing, 3.75 kHz or 15 kHz, the IFFT size equals 512 or 128, respectively. It should be noted that the size of the IFFT processing directly influences the computational cost of the module. However, considering the fact that, due to 12 or 48 subcarrier-based symbol allocation, the input vector contains a lot of zero data (due to the zero-padding operation). It is also a good example in the context of an optimization task by reducing the number of the IFFT calculations regarding unused transformation elements.

#### 5.8.3. Cyclic Prefix Attachment

The last function of the OFDM module is the CP attachment. The main concept of this block represents the process of replicating the end of each symbol at the beginning of the same symbol. The exact number of CP samples may be obtained based on the time dependencies presented in Table 3 and 1.92 MHz sampling frequency. Thus, the CP length for the 3.75 kHz subcarrier spacing equals 16 samples, and for 15 kHz: 10 samples (every first symbol in a slot) or 9 samples (the other six symbols in a slot). Moreover, the additional frequency shift should be applied to provide the phase continuity of the samples.

## 6. Analysis of the Results and the Use Cases

In this section, the benchmark tests of the NPUSCH module of the NB-IoT uplink path are presented. It is one of the possible framework realizations which could be treated as the starting point for the students to work on their own implementations. The framework modules are set to generate a set of complex samples at the output, i.e., the IQ waveform of the radio signal with the 1.92 MHz sampling frequency (compatible with, e.g., SDR USRP devices). Computations were performed on the PC class computer with the Intel Core i7-10700 2.9 GHz processor and 32 GB of DDR4 type RAM. However, as previously mentioned, the framework can be easily executed and investigated on various hardware platforms. Thus, to present results in a more universal and useful way, the normalized times (by means of the total computation time) for sequential processing are presented, where the measurement accuracy was 1 μs. It should be noted that the framework must operate in real time, which means that cumulative preparation time of one 10 ms radio frame must be shorter than the frame itself.

For the benchmark purposes, the prepared input sensor data were generated randomly with uniform distribution, but the TBS ranges were adjusted to maintain the constraints described in the standard. It should be noted that, as an original part of the provided framework, authors share the package for each module or decomposed function, i.e., the input and output vectors with additional parameters of the test matrix that are available under the terms of the CC BY license for educational purposes. At this point, it is worth mentioning that the presented simulation studies were carried out for different sizes of the $N_{RU}$, $N_{slots}^{UL}$ and $N_{SC}$ to obtain the results that will correspond to the real 3GPP-based NB-IoT network cases. In the real NB-IoT user terminal, the transmission parameters are received from the eNB in the broadcast channel within the master information block (MIB) and system information block (SIB) messages received by the sensor device before the communication. However, for research and teaching purposes, they can be generated separately for different student groups.

During the didactic use cases, the data streams can be adjusted, e.g., to the real operation of the data sensors or even show the dependencies how the propagation conditions determine the transmission parameters and the final energy usage of the sensor data transmission. It means that data can be generated more frequently (divided into smaller TBS [38]) for the sensor devices which monitor environmental parameters that change dynamically, e.g., wind speed, or with large time intervals. For the purpose of the sample analysis presented in the article, configurations with all the sizes of the available TBSs were checked, which corresponds to more than 1000 transmissions with a length of 1 frame up to 39 frames.

**Figure 7:**
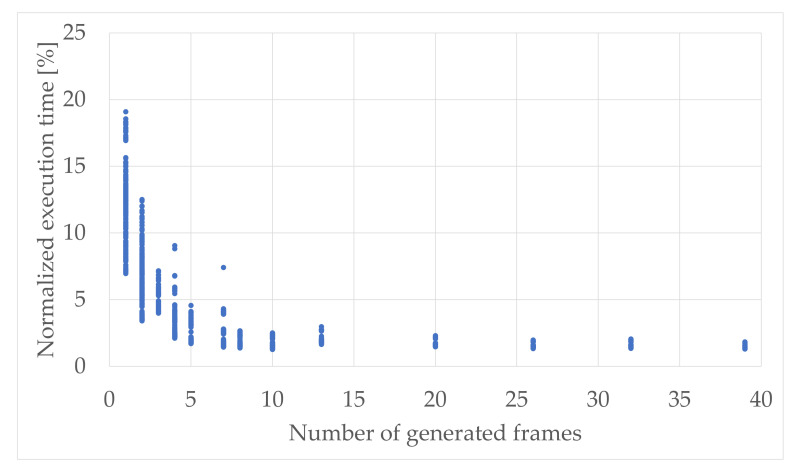
Normalized execution time of all the modules except the OFDM generation as a function of the generated number of radio frames.

Taking into consideration the character of the computations, such as formulation of the radio frames and the complex signal processing, it was expected that the OFDM generation functional block (Section 5.8.2) will be the one which is the most time-consuming. Thus, it was decided to firstly analyze the proportions of execution time between this module and the rest of the processing chain as a function of the output radio signal length. In Figure 7, the normalized execution time of all the modules, except the OFDM generation, as a function of the generated number of radio frames are shown.

As expected, by means of procedural single-thread usage, the disproportion between the OFDM module and the sum of execution times of other modules can be clearly seen. The dependency between the execution time is exponential rather than linear in this case, where minimum value was 1.3% and maximum value reached 19.1%. It means that the impact in the total uplink efficiency of the blocks from CRC module to the mapping to physical resources increases for the good propagation conditions, which results in using the multitone transmission that is available only in the Δf= 15 kHz subcarrier spacing variant. Thus, the 128 point FFT and IFFT transformations are used rather than the 512 point one, which, with the less number of matrix operations during OFDM symbols formulation, leads to more meaningful impact in that block chain.

**Figure 8:**
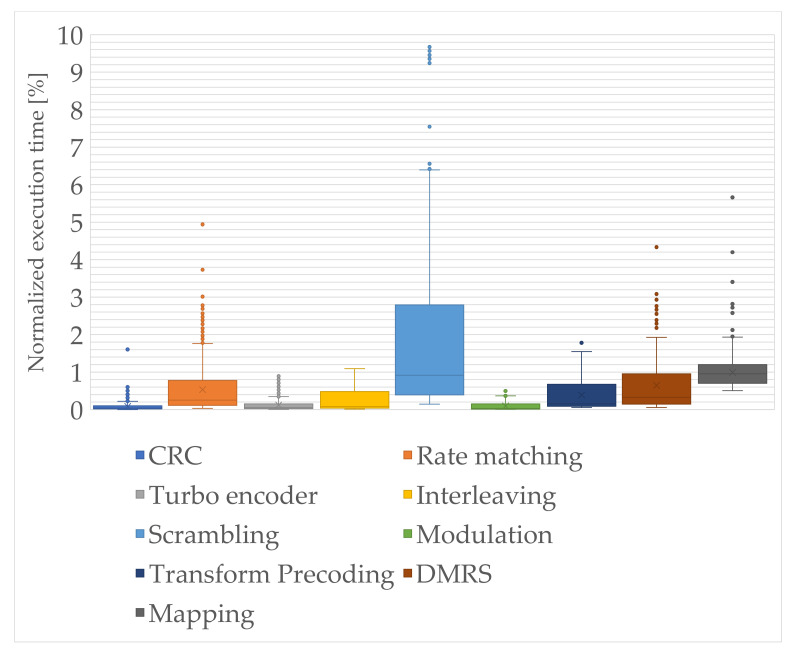
Normalized execution time of modules in the block chain between CRC and mapping to physical resources.

**Figure 9:**
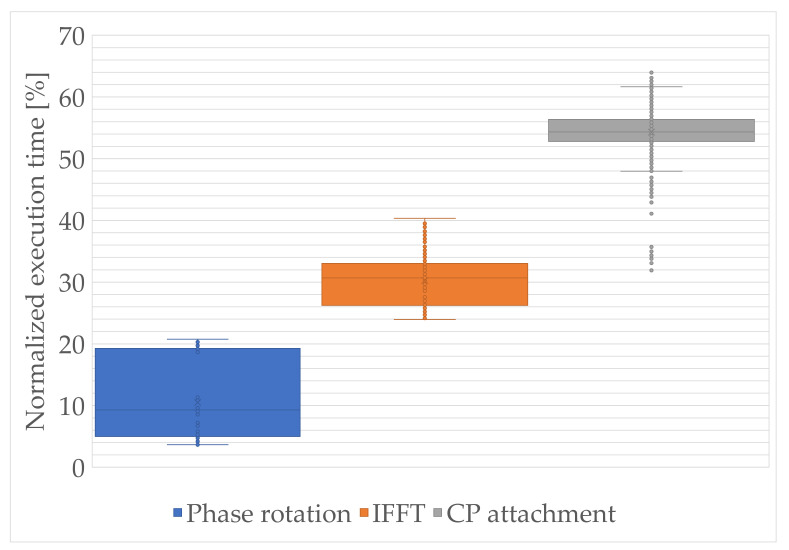
Normalized execution time of the OFDM generation decomposed functions.

Based on the obtained results, especially from the educational point of view, it is important to learn the relations between the decomposed sections of the modules. In Figure 8 and Figure 9, the decomposed functional sections and their normalized execution times, with respect to the total execution times of the whole framework, are presented as box-and-whisker plots. It consists of the boxplot limited by the first quartile (25th percentile), median, and third quartile (75th percentile) values, and whisker that points to the minimum and maximum values. However, the minimum and maximum values are the lowest and highest data points excluding the outliers, which were marked as additional dots in the figures. In addition, in Table 8, the calculated reference normalized execution time results including the outliers are presented, for each integrated or decomposed module.

The NB-IoT scrambling, i.e., the pseudorandom sequence generation for the NPUSCH formulation, is one of the most computationally intensive modules in this chain. In this module, the sequence which is further used for generating the scrambling sequence and scrambled with the input code word must be initialized. As it can be seen, the bitwise operations, if properly implemented even with the use of procedural C/C++ language, are more efficient than operations on the floating point values. However, modules that occur after the scrambling one, even when they are operating on the floating point complex signals, are still processed faster. This results from the effectiveness of the low-leveled memory blocks copying and performance of the FFT library [39] with assembler parts. It should be also noted that the DMRS module uses the same scrambling methods as in the scrambling module. However, the difference is in the output of the scrambling base module. The output length is based on the $N_{RU}$ and $N_{slots}^{UL}$ and the number of repetitions, which results in a much higher amount of data for analysis, with respect to the DMRS sequences that can be copied efficiently into the proper RUs.

This section of the uplink signal processing can be efficiently implemented in the FPGA, especially taking into consideration the bitwise operations and the parallel processing ability. This can be crucial, especially in the harsh propagation environments where the number of security mechanisms needed to be used in order to provide the quality of service (QoS) in the wireless sensor networks (WSNs) is higher. In addition, such approach of duplicating the modules in the hardware is highly desirable from the educational point of view. Learning the process of designing and developing the proper projects may show a great advantage of using the FPGA in the signal processing performed in the dynamically reconfigurable remote radio heads in the LTE eNB and the 5G New Radio Next Generation NodeB (NR gNB), and also enable the use of practical solutions based on the theoretical description.

On the other hand, as was previously presented, the OFDM generation module is computationally demanding, mainly due to the numerous matrix operations. Each OFDM symbol preparation is based on the IFFT processing and the CP attachment, which is essential in providing the orthogonality. Basing on the knowledge concerning CP attachment (a theoretical copying), the highest execution times can be misleading and should be definitely explained for the purposes of the teaching process. In general, the approach of just copying and appending the last part of the OFDM symbol onto the front of it is not the only process that is performed in that module. In addition, there is a need to maintain the phase continuity between successive samples by shifting the frequency of the signal by half of the subcarrier spacing values (for ensuring spectrum symmetry with respect to the DC component) and performing window function for the PAPR control. The process of designing the proper filtration of the signal at the output is yet another important case. PAPR minimization can be crucial, especially in the context of green sensors utilization and power efficiency, and thus it may be a good opportunity to include practical aspects and a learn-by-practice approach in the educational process.

The IFFT operation, with the length of 128 or 512 points (dependent on the subcarrier spacing) in the NB-IoT interface, may be asymmetrically reduced and/or processed parallel, which determines designing and development of the multithreaded environment. These are the potential topics of the students’ research activity in the Masters or Bachelors degrees, either during laboratories or qualification works. The phase rotation module, based on its description introduced in Section 5.8.1, can also be optimized by the usage of the parallel processing and exponential values tables for the proper configurations in the device static memory.

## 7. Conclusions

The complexity of the cellular systems requires wide knowledge on the radiocommunication signal processing. The new era of the technical sciences, including radiocommunication, is mostly based on the software solutions. Such an approach enables simulations in the different types of environment, from the very basic to the harsh ones, or different use cases. It gives scientists and students opportunities to develop best-suited cellular concepts, such as the NB-IoT. In Section 3, some NB-IoT simulators were described in terms of their abilities and limitations. However, the implementation of the real radiocommunication interface in the sensor devices on the basis of computer simulations is often very ineffective, as it does not take into account the limitations of the low-level implementation in general purpose processors or the FPGA matrices. Therefore, the implemented didactic framework, based on the didactic experience of the authors, goes beyond the simple simulations concerning implementation constraints as well. The main advantage of this approach is the opportunity to observe and further analyze both inter- and intramodule data flow along the whole physical layer, according to Figure 2. It is worth mentioning that this feature is not provided by the commercially available simulation tools such as Matlab or Simulink. All presented functions are organized as the functional modules that are compliant with Rel. 13/14 3GPP standards. Their detailed description is included in Section 5, and further supported by the functional analysis included in Section 6.

Not every module has the same computational cost, so it is reasonable to focus on the software optimization as it directly influences the power consumption when the computational time is reduced. From the educational point of view, it is even more important to implement some software parts step by step, because it provides better understanding of the issue. Observations presented in Section 6 allow students to assess which part of the uplink they should expend more effort on, especially in the context of efficient sensor networks. It is worth mentioning that every part of the PHY layer can be checked and evaluated independently by using the specially generated sets of the input vectors. During the future works, authors want to propose a similar framework for the downlink path that is currently under development. It will provide similar possibilities which will extend the didactic abilities of the hardware- and software-based laboratory for the Bachelors and Masters students.

## Figures and Tables

**Table 1:** Intercolumn permutation pattern for sub-block interleaver.

Number of Columns	Intercolumn Permutation Pattern
$C_{subblock}^{TC}$	<$P\left(0\right),P\left(1\right),...,P\left(C_{subblock}^{TC}-1\right)$>
32	<0, 16, 8, 24, 4, 20, 12, 28, 2, 18, 10, 26, 6, 22, 14, 30, 1, 17, 9,
	25, 5, 21, 13, 29, 3, 19, 11, 27, 7, 23, 15, 31>

**Table 2:** Block encoder 1/16.

NPUSCH Format 2 Bit	Output Codeword
0	<0, 0, 0, 0, 0, 0, 0, 0, 0, 0, 0, 0, 0, 0, 0, 0>
1	<1, 1, 1, 1, 1, 1, 1, 1, 1, 1, 1, 1, 1, 1, 1, 1>

**Table 3:** NB-IoT parameters [2].

Subcarrier Spacing	$N_{sc}^{UL}$	$T_{slot}$	CP (Cyclic Prefix) Length
Δf= 3.75 kHz	48	2 ms	8.33 μs
Δf= 15 kHz	12	0.5 ms	symbol #0: 5.2 μs
			symbols #1–6: 4.7 μs

**Table 4:** NPUSCH parameters [2].

NPUSCH Format	Δf	$N_{\mathrm{sc}}^{\mathrm{RU}}$	$N_{\mathrm{slots}}^{\mathrm{UL}}$
1	3.75 kHz	1	16
	15 kHz	1	16
		3	8
		6	4
		12	2
2	3.75 kHz	1	4
	15 kHz	1	4

**Table 5:** BPSK modulation [2].

b(i)	I	Q
0	$1/\sqrt{2}$	$1/\sqrt{2}$
1	$-1/\sqrt{2}$	$-1/\sqrt{2}$

**Table 6:** QPSK modulation [2].

b(i), b(i+1)	I	Q
00	$1/\sqrt{2}$	$1/\sqrt{2}$
01	$1/\sqrt{2}$	$-1/\sqrt{2}$
10	$-1/\sqrt{2}$	$1/\sqrt{2}$
11	$-1/\sqrt{2}$	$-1/\sqrt{2}$

**Table 7:** NPUSCH modulation schemes [2].

NPUSCH Format	$N_{\mathrm{sc}}^{\mathrm{RU}}$	Modulation Scheme
1	1	BPSK, QPSK
	>1	QPSK
2	1	BPSK

**Table 8:** Normalized execution times of the framework modules and decomposed functions.

	Min	Quartile 1	Median	Quartile 3	Max
CRC	0.01	0.02	0.05	0.1	1.61
Rate matching	0.03	0.11	0.26	0.78	3.73
Turbo encoder	0.01	0.03	0.06	0.15	0.92
Interleaving	0.01	0.04	0.08	0.48	1.09
Scrambling	0.15	0.39	0.92	2.79	9.67
Modulation	0.01	0.02	0.03	0.09	0.5
Transform precoding	0.05	0.09	0.15	0.68	1.78
DMRS	0.06	0.14	0.33	0.95	4.34
Mapping	0.5	0.7	0.96	1.2	5.66
Phase rotation	3.65	5.0	9.3	19.2	20.8
IFFT	23.9	26.2	30.7	33.0	40.3
CP attachment	31.9	52.8	54.3	56.4	64.0

## Data Availability

Input and output vectors with additional parameters test matrix are available for free from the authors with the CC BY license for educational purposes.
